# Molecular mechanisms of coronary microembolization-induced MINOCA

**DOI:** 10.1007/s00395-026-01189-2

**Published:** 2026-07-04

**Authors:** Jasper Iske, Henriette Thau, Joshua M. Mesfin, Christien M. Beez, Petra Wolint, Jonas Hildinger, Nicolas Musigk, Timo Z. Nazari-Shafti, Adam Penkalla, Volkmar Falk, Bettina Heidecker, Bertil Lindahl, Maximilian Y. Emmert, Nikola Cesarovic

**Affiliations:** 1 Department of Cardiothoracic and Vascular Surgery, Deutsches Herzzentrum Der Charité (DHZC), Berlin, Germany; 2 Charité – Universitätsmedizin Berlin, Corporate Member of Freie Universität Berlin and Humboldt-Universität Zu Berlin and Berlin Institute of Health, Berlin, Germany; 3 Berlin Institute of Health at Charité-Universitätsmedizin Berlin, Berlin, Germany; 4 DZHK (German Centre for Cardiovascular Research), Partner Site Berlin, Berlin, Germany; 5 BIH Center for Regenerative Therapies (BCRT), Berlin Institute of Health at Charité-Universitätsmedizin Berlin, Berlin, Germany; 6 Department of Health Sciences and Technology, ETH Zürich, 8092 Zürich, Switzerland; 7 Department of Cardiology, Angiology and Intensive Care, Deutsches Herzzentrum Der Charité Berlin (DHZC), Berlin, Germany; 8 Michael E. DeBakey Department of Surgery, Baylor College of Medicine, Houston, TX USA; 9 Department of Medical Sciences, Cardiology, Uppsala University, Uppsala, Sweden; 10 Uppsala Clinical Research Center, Uppsala University, Uppsala, Sweden; 11 Institute for Regenerative Medicine (IREM), University of Zürich, Zurich, Switzerland

**Keywords:** Myocardial Infarction, MINOCA, Coronary microembolization, Biomarker

## Abstract

Compared to classic myocardial infarction (cMI), myocardial infarction with non-obstructive coronary arteries (MINOCA) is characterized by symptoms consistent with acute coronary syndrome, but without demonstrable coronary obstruction. Therefore, its diagnosis remains challenging and often relies on cardiac magnetic resonance imaging (MRI), which is usually performed only days after the index event, leading to delayed diagnosis and initiation of therapy. Coronary microembolization (CME) has been described as one of the major pathologies underlying MINOCA. However, the molecular mechanisms of CME remain poorly understood, resulting in a lack of rapid diagnostic tools and specific therapy approaches. In this review, we describe the molecular underpinnings of CME-derived MINOCA: an inflammatory environment inducing significant cellular damage that is conserved between cMI and MINOCA. While we note that some of these inflammatory and cellular death pathways are shared with cMI, we dissect mechanistic distinctions in CME-derived MINOCA such as dysregulated immune responses and higher miRNA activity. Most importantly, as molecular treatments for MI are currently in late-stage clinical trials, we can thus differentiate which therapeutics may work better for MINOCA. Thus, these findings can be used as a roadmap for diagnostic biomarkers and targeted therapeutic approaches for MINOCA.

## MINOCA is a subset of MI with unique clinical presentations

Despite major advances in the treatment of acute myocardial infarction (AMI), myocardial infarction without obstructive coronary arteries (MINOCA) remains a clinically challenging diagnosis. It is estimated that a working diagnosis of MINOCA accounts for between 6 and 8% of all cases of myocardial infarction (MI) [141, 191]. Interestingly, however, only around 24% of patients with this diagnosis show features consistent with subendocardial infarction; the remainder show signs of various forms of cardiomyopathy [142], further highlighting the clinical challenge of this diagnosis. In contrast to classic myocardial infarction (cMI), in which a coronary vessel is obstructed by a large occlusion, a proportion of patients with MINOCA suffer from many, small microvascular occlusions, which are not detected by cardiac catheterization [155]. Patients with MINOCA can present with a very similar clinical picture as cMI [38]. However, MINOCA is associated with non-specific electrocardiogram changes occurring in predominantly younger, female patients with fewer cardiovascular risk factors [98, 142, 157]. Considering its challenging diagnosis, MINOCA is associated with a relatively high risk of major adverse cardiac events leading to early rehospitalization. As a result, patients with MINOCA incur 1-year mortality and reinfarction rates of 3.4% and 2.6%, respectively [143].

The diagnosis of MINOCA remains challenging and often relies on cardiac magnetic resonance imaging (CMR), which is typically performed days/weeks after the index event, leading to delayed diagnosis and initiation of therapy. CMR is the most specific diagnostic modality, but is difficult to implement in the acute setting. Consequently, it has been shown that MINOCA patients are significantly less likely to receive cardioprotective medications when compared to patients with cMI, possibly due to the lack of appropriate early diagnostic tools [2]. In addition, due to insufficient characterization of the pathophysiological processes underlying this disease [130], no specific therapy has been identified [146]. Therefore, there is an urgent and unmet medical need to characterize the pathophysiology of MINOCA as distinct from cMI to identify key disease driving processes that can be used to develop rapid diagnostic tools to enable timely diagnosis and initiation of therapy. In addition, such insight into disease-specific molecular mechanisms may reveal novel therapeutic targets. This review aims to delineate the molecular mechanisms underlying MINOCA, to characterize tissue and cell damage, as well as to identify and compare the inflammatory patterns of MINOCA and cMI.

## Coronary microembolization models constitute MINOCA pathology

MINOCA is prevalent in patients displaying non-ST-segment-elevation myocardial infarction (NSTEMI) [10]. In the last decade, there has been a significant increase in acute coronary syndromes with no ST elevation deriving from plaque erosion or rupture [90]. The association between NSTEMI and plaque erosion has highlighted coronary microembolization (CME) as a pathologic mechanism [90]. Notably, plaque erosion and subsequent CME represent the major pathology in 40% of all MINOCA cases [139, 175]. CME can also occur as a result of cardiac interventions such as percutaneous coronary intervention (PCI) [90], in which thrombotic or atherosclerotic material is dislodged into the cardiac microcirculation. Embolization of the coronary microcirculation promotes myocardial necrosis [6, 138] and thus leads to post-PCI microvascular obstruction (MVO) [99].

**Fig. 1:**
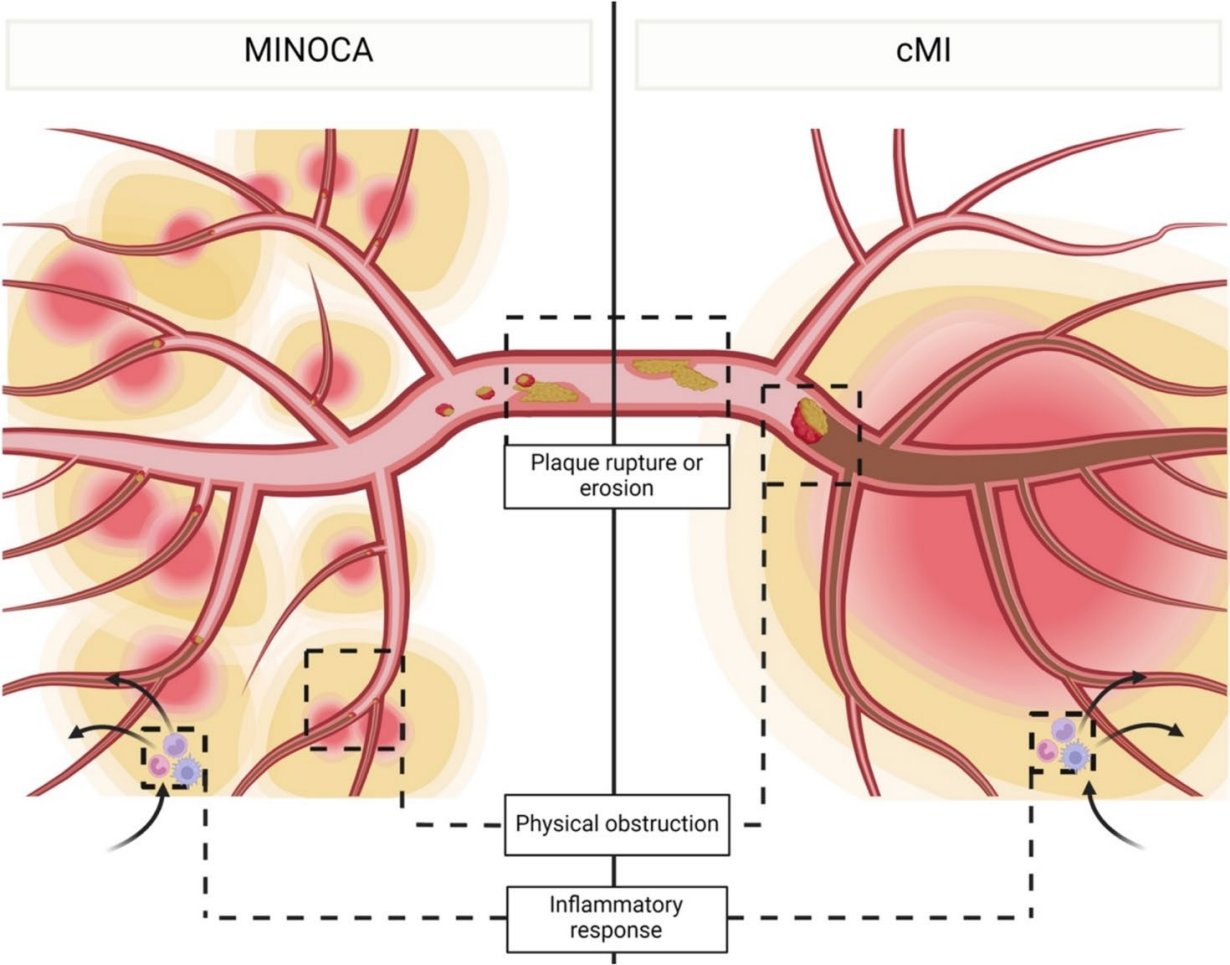
Schematic overview of the process and subsequent tissue damage in MINOCA and cMI. In both myocardial infarction with non-obstructive coronary arteries (MINOCA) and classic myocardial infarction (cMI), plaque builds up in the arteries. In MINOCA, the plaque erodes, forming multiple microthrombi that occlude the smallest coronary capillaries, leading to oxygen deprivation in many small areas. Subsequent infiltration of inflammatory cells into the damaged tissue leads to increased expression of tumor necrosis factor *α* (TNF-*α*) and generation of reactive oxygen species (ROS), ultimately resulting in a profound inflammatory response. In cMI, plaque rupture leads to the formation of a thrombus that subsequently occludes one of the coronary arteries, resulting in an oxygen deficiency in the downstream tissue. The prolonged ischemia leads to necrosis of the myocardium, resulting in an inflammatory response mediated primarily by the release of TNF-*α*. Created with *BioRender.com*

The initiating event in CME is typically rupture or erosion of atherosclerotic plaques [67] (Fig. 1). While the debris causes physical occlusion of coronary microvessels, the released soluble debris is flushed into the coronary microcirculation and triggers processes such as platelet aggregation, endothelial dysfunction, and vasoconstriction [68, 90]. First described by Davis and Falk, microemboli composed of fibrin, platelet aggregates, hyaline, and cholesterol were observed at autopsy of patients with coronary artery disease who died of sudden cardiac death [90, 162].

Beyond physical MVO, the vasoconstrictor response to CME constitutes a distinct and clinically important pathophysiological component. Microembolic debris releases soluble mediators, such as thromboxane A2, endothelin-1 and serotonin derived from activated platelets, which can thus induce vasoconstriction in obstructed and adjacent non-obstructed microvasculature. This phenomenon can thus amplify the perfusion deficit beyond the initial, physical occlusion [100]. Here, the vasoconstrictor response has been shown to contribute independently to the perfusion-contraction mismatch pattern, suggesting that coronary vasomotor dysfunction could be a primary consequence of microembolization [89]. Interventions that solely target the thrombotic CME component may leave the vasoconstrictor-mediated perfusion unaddressed, leading to residual contractile dysfunction. Thus, vasodilatory agents may offer dual mechanistic benefit through vasoconstriction and inflammation reduction, demonstrating their potential as an independent therapeutic agent.

**Fig. 2:**
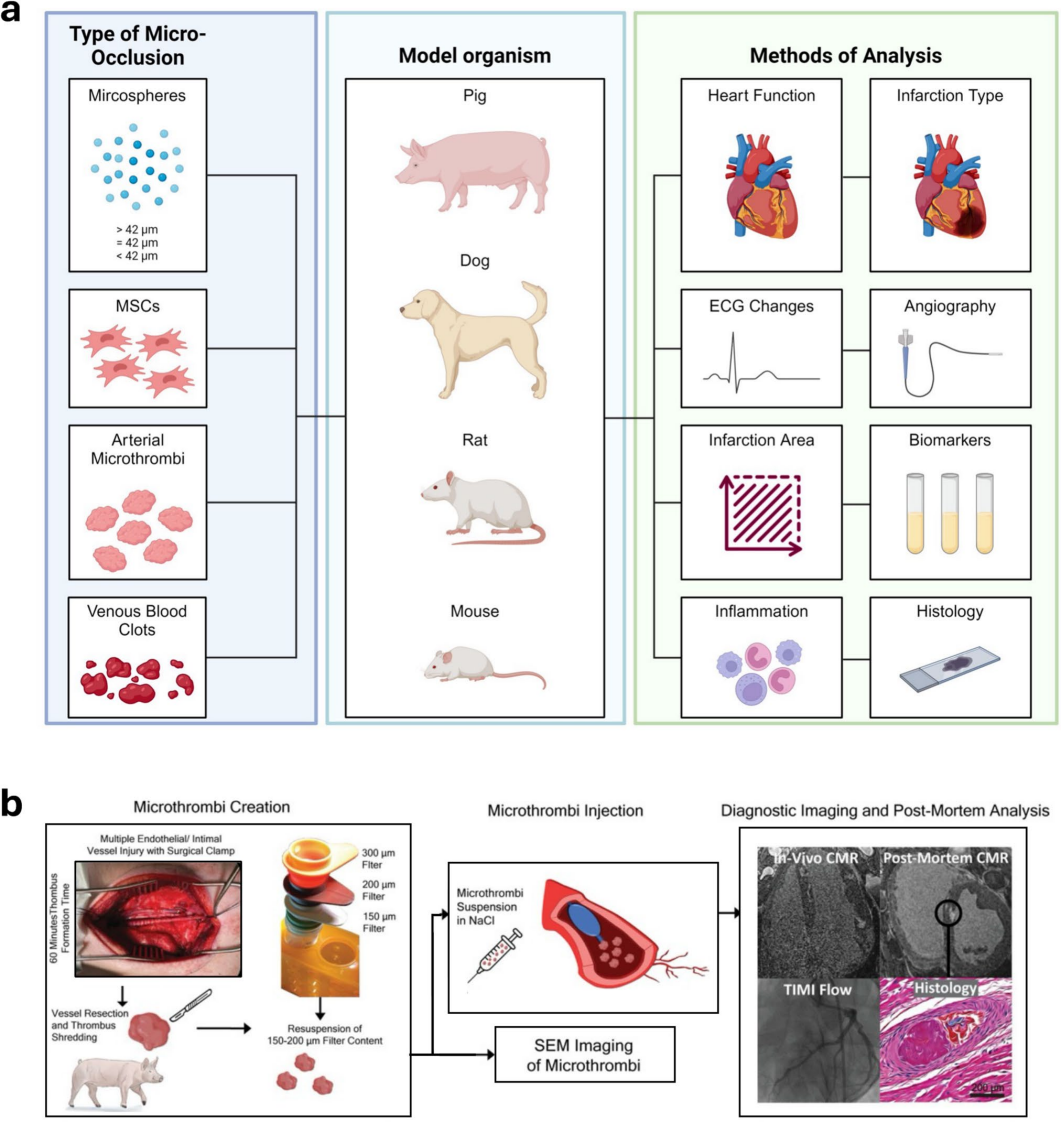
Overview of myocardial microocclusion models. **a** Various microsphere sizes, mesenchymal stem cells (MSCs), arterial microthrombi, and venous blood clots all play a role in microocclusion. With various model organisms in mind, depending on the experimental context, typical methods of analysis are done to measure overall cardiac function, mechanisms of injury, and particular markers to leverage for microocclusion treatment. Created with *BioRender.com*** b** Autologous microthrombi administration is the most translationally relevant model for microocclusion. Adapted from Cesarovic et al*.* [28]

To study CME mechanisms more effectively, multiple small (mouse and rat) and large (porcine) animal models have been developed with the goal of observing the mechanism and effects of microembolization on cardiac functionality (Fig. 2). While CME has been induced through injection of polystyrene microspheres, these microspheres do not exhibit pro-inflammatory and pro-thrombotic properties due to their inert nature [90]. The severity of CME-derived microembolization is greatly dependent on microparticle size and number, with varied particle sizes (10–100 µm). Uniquely, administration of additional or larger particles does not induce hyperemia. Instead, novel approaches involving the application of homologous or autologous thrombus material around 100–200 µm allow for a more accurate simulation of CME, with a recent animal model capturing all clinical aspects of MINOCA [28, 90].

While animal models have provided substantial mechanistic insight into CME pathophysiology, using human biological material, such as isolated human coronary vessels and plasma-based assays, can offer translational fidelity without full use of in vivo systems. Ex vivo human coronary artery preparations have been used to test endothelial dysfunction, vasoconstrictor responses, and platelet–endothelial interactions under conditions mimicking microembolic stress [44, 88, 91, 128]. In addition, with patient-derived material from CME or MINOCA, plasma-based models have been used to assess platelet aggregation dynamics, coagulation cascade activation, and cytokine release profiles in a controlled environment [75, 111]. Here, these approaches allow for the use of human tissue with preserved genetic and phenotypic diversity, which reduces concern over species-specific artifacts present from rodent or porcine models. However, ex vivo vessels lack the systemic neurohormonal environment, do not recapitulate coronary perfusion pressure dynamics, and cannot model embolic events characteristic of CME. Plasma models also fail to capture the spatial and temporal complexity within CME-derived MINOCA. Thus, human vessel and plasma models exist as complements to in vivo work, rather than replacements, with their integration strengthening future CME research pipelines and thus the translational relevance of preclinical findings.

Unlike epicardial coronary artery occlusion, microembolized myocardium shows a perfusion–contraction mismatch pattern leading to coronary blood flow fluctuation [49, 162]. Microsphere injection in animal models demonstrated an immediate decrease in coronary blood flow followed by elevated blood flow, a phenomenon known as reactive hyperemia [136, 163]. Microembolization results in adenosine release from the affected tissue, which dilates vessels in the surrounding area [73]. The changes in contractile dysfunction follow a defined pattern of three phases: (i) an immediate decrease after microembolization, (ii) a partial recovery of contractile function, and (iii) a deterioration 6–12 h following microembolization [90]. Similar to blood flow, the response of global left ventricular (LV) function is also dependent on the size and area of the affected myocardium with reduced perfusion [90]. With repeated CME, baseline coronary blood flow may remain normal or even exceed the normal value, but adenosine-responsive coronary reserve is significantly reduced [163]. This reduction is caused by both reactive hyperemia and a decrease in maximal blood flow due to physical obstruction of the coronary microvessels [163].

Apart from a decrease in contractile function, CME models reveal edema, patchy perfusion defects, and microinfarcts, which are further reflected within histology [19, 26, 133, 152]. Caused by physical obstruction of functional end arteries, microinfarct size area is correlated with the site of vascular obstruction as well as emboli diameter [54]. In addition to edema, endothelial sloughing has been seen within the intramyocardial capillaries [153], where histologic changes after CME include coagulative necrosis [63, 114] with loss of nuclei, narrow and elongated myofibers, and myocardial striation [114]. Finally, immune cell aggregates, primarily composed of macrophages, comprise the subendocardial microinfarction area in pigs [152], miniswine [114], and rats [63]. In multiple animal models, the microinfarction site is characterized by an infiltration of leukocytes, such as monocytes and macrophages [90, 106, 162]. Given the small infarcted area and no persistent macroscopic decrease in blood flow after microembolization, the inflammatory response appears to be the main drive behind the loss of regional contractility [162].

A critical limitation of existing CME animal models is their failure to recapitulate the metabolic background present in human MINOCA patients. As the majority of clinical MINOCA patients have existing cardiovascular risk factors, such as obesity, insulin resistance, dyslipidemia, and hypertension, the combination of these factors constitutes metabolic syndrome [173], which is present on a polygenic background in humans. Standard rodent and porcine CME models are induced in metabolically normal animals. However, the Ossabaw minipig represents the most clinically relevant large animal model for cardiovascular research, as it can develop diet-induced obesity, insulin resistance, hypertension, and dyslipidemia on a polygenic background [52, 94]. Among rodent models, the Nile rat [135] and sand rat [85] develop spontaneous type 2 diabetes and metabolic dysregulation under standard dietary conditions [97]. As metabolic syndrome can independently promote endothelial dysfunction, heightened thrombogenicity, and exaggerated inflammatory responses, recapitulating this clinical feature is critical towards understanding the response severity in the clinical MINOCA population.

In addition to immune cell fluctuations, the CME inflammatory response has been associated with increased expression of tumor necrosis factor *α* (TNF-*α*) in macrophages and cardiomyocytes, along with increased levels of interleukins (ILs) and inducible nitric oxidase (iNOS) [90]. The major inflammatory marker TNF-*α* plays a critical role in promoting tissue inflammation, leukocyte infiltration, and dysregulating contractile function [5, 90]. TNF-*α* upregulation in response to CME induction may be due to either an autocrine or paracrine response between macrophages and cardiomyocytes present in the infarcted and non-infarcted myocardium [162]. This suggests that the interaction between infarcted regions and the surrounding viable myocardium, alongside the resulting inflammatory response, has a greater impact on contractile function than the extent of actual cell loss within the ischemic tissue [125]. Finally, to demonstrate that TNF-*α* expression is necessary for myocardial contractile dysfunction, TNF-*α* infusion without prior microembolization triggered a comparable response to microembolization [50]. In addition, treatment with anti-TNF antibodies annihilated CME-induced contractile dysfunction [50].

TNF-*α* has also been shown to activate a signaling cascade involving nitric oxide (NO) and reactive oxygen species (ROS), both of which are implicated in TNF-α-induced dysfunction [15, 162]. In general, ROS formation has been shown to induce myocardial damage at high concentrations [51] and lead to subsequent oxidation of myofibrillar proteins [24]. ROS formation has been characterized as an additional mechanism linking the inflammatory response to the occurrence of contractile dysfunction. The oxidation of tropomyosin might represent an end effector of the transduction pathway triggered by CME that links the inflammatory response to the failure of contraction [24]. ROS-mediated oxidative modification of troponin I reduces myofilament calcium sensitivity, which represents a distinct downstream mechanism where CME-induced oxidative stress translates into contractile dysfunction. Here, ROS represents a mechanistic bridge between the inflammatory response and cardiac functional impairment in CME-induced MINOCA [160]. Finally, TNF-*α* expression and ROS formation have been shown to amplify expression of each other, resulting in further oxidative stress and the inflammatory responses to CME [162].

In addition, NO production following CME exhibits a biphasic, temporal release. Within minutes post-embolization, endothelial nitric oxide synthase is released. Inducible nitric oxide synthase is then upregulated over the next few hours, leading to sustained contractile dysfunction [159, 174]. Sphingosine has also been identified as an upstream mediator of TNF-*α* signaling in CME-induced myocardial dysfunction [174], resulting from ceramide hydrolysis downstream of TNF receptor activation and acting through mechanisms partially independent of NO and ROS signaling [159]. Thus, sphingosine impairs myofibrillar calcium sensitivity and contributes to contractile depression [159]. Here, sphingosine, nitric oxide, and reactive oxygen species provide a definitive link from the inflammatory response to contractile dysfunction in CME, which suggests that a therapeutic strategy targeting one aspect of the network may not lead to full functional recovery.

**Fig. 3:**
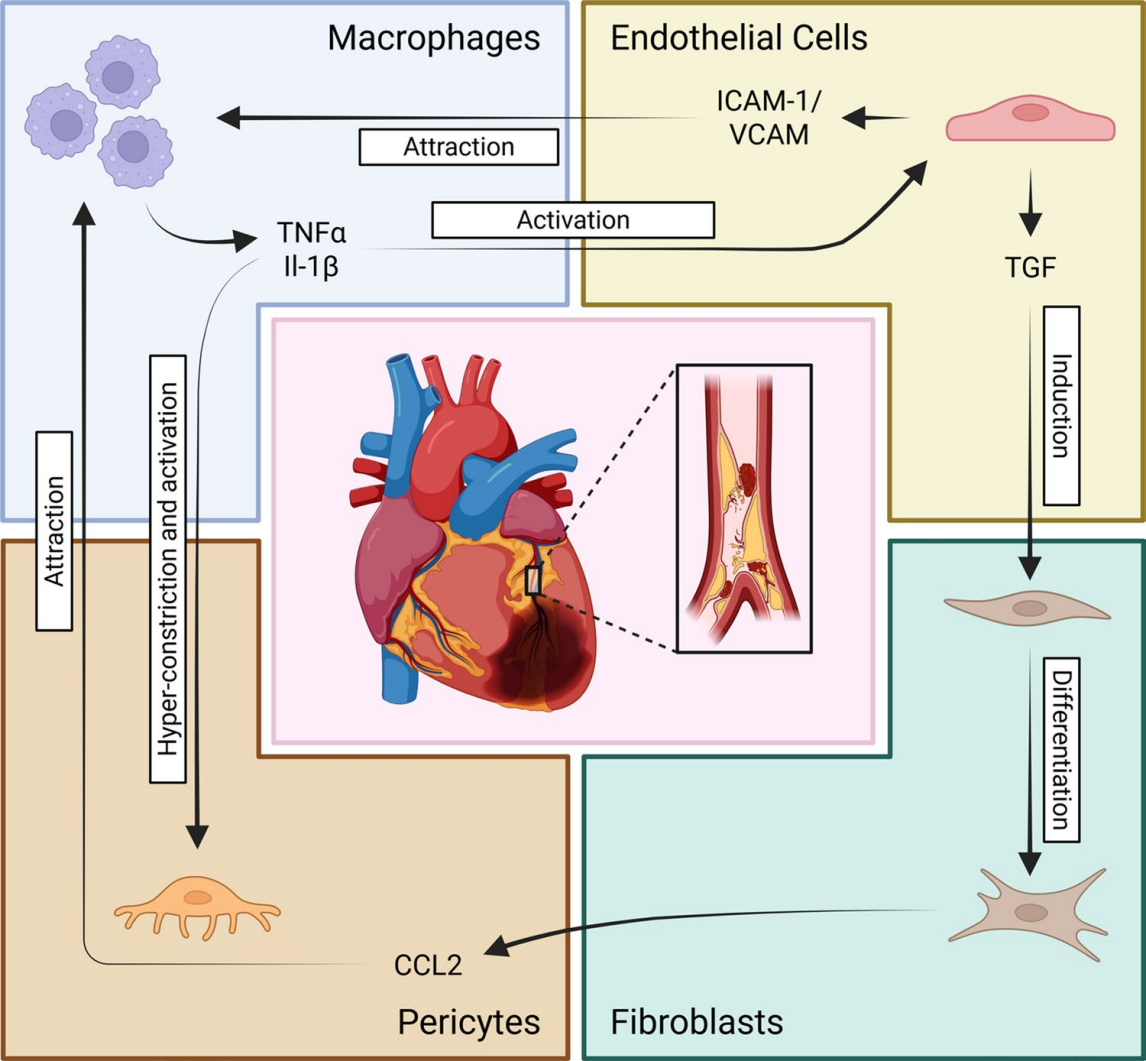
Complexity of cell-to-cell communication and cell-type-specific signaling networks in classic myocardial infarction. Schematic representations of cell-to-cell interactions in cMI. Ischemia activates macrophages, which secrete factors such as TNF*α* and IL-1β. This initiates a cascade in which endothelial cells respond to macrophage-derived inputs by upregulating cell adhesion molecules. This drives the rolling, adhesion, and attraction of additional circulating monocytes. Meanwhile, activated endothelial cells secrete TGF-β, leading to the differentiation of myofibroblasts from otherwise quiescent cardiac fibroblasts. Myofibroblasts then secrete other proinflammatory factors, such as CCL2, which establishes a potent chemotactic gradient. In parallel, macrophage-derived TNF*α* and IL-1β induce acute hyperconstriction and microvascular activation of microvascular basement membrane-resident pericytes. This contributes to the "no-reflow" phenomenon within the infarct core. Similar cellular patterns must be investigated in CME-induced MINOCA to elucidate potential differences. Created with *BioRender.com*

While the cell–cell interactions in cMI are relatively well characterized (Fig. 3), a recurring limitation in mechanistic CME-induced MINOCA characterization is the lack of cell-specific resolution with respect to signaling data. Previous studies have evaluated upregulation of inflammatory mediators such as TNF-*α* via bulk methods, and have identified macrophages and monocytes as the principal source of TNF-*α* following CME induction in porcine models, with TNF-*α* expression detectable in both the infarcted and peri-infarct myocardium [162]. However, it is unknown how other cell types, such as cardiac fibroblasts, endothelial cells, and pericytes, contribute toward CME pathophysiology. Future studies employing single-cell RNA sequencing or spatial transcriptomics in CME models would advance cell-type mechanistic understanding and inform future therapeutic targets in CME-induced MINOCA.

With all these pathological and clinical presentations of CME-induced MINOCA in mind, the molecular response of CME-induced MINOCA still remains largely unknown due to diagnostic difficulties. Thus, we will compare the molecular expression profiles of CME-induced MINOCA (hereafter referred to as 'MINOCA') with cMI, which is highly characterized at the molecular, cellular, and proteomic levels. Through these comparisons, underlining these potential differences may be used to determine novel MINOCA biomarkers (Table 1).

**Table 1:** Comparison of the expression of various possible biomarkers in MINOCA/CME and cMI. *BAX* Bcl-2 associated X-protein, *Bcl-2* B-cell lymphoma 2, *XIAP* X-linked inhibitor of apoptosis protein, *LOX-1* lectin-like oxidized low-density lipoprotein receptor-1, *TNF-α* tumor necrosis factor *α*, *IL* interleukin, *HMGB1* high mobility group box 1, *NFκB* nuclear factor kappa B, *iNOS* inducible nitric oxide synthase, *Egr-1* early growth response 1, *PTEN* phosphatase and tensin homolog

Marker	MINOCA/CME	cMI	Cellular process	References
BAX	↑	↑	Apoptosis	[93, 118, 144]
Cytochrome c	↑	↑	Apoptosis	[3, 53, 118]
Cleaved caspases	↑	↑	Apoptosis	[18, 93, 118]
Bcl-2	↓	↓	Anti-apoptotic	[93, 110, 144]
XIAP	↓	↓	Anti-apoptotic	[37, 196, 199]
LOX-1	↑	↑	Inflammation, Apoptosis	[11, 72, 102, 118, 193]
TNF-*α*	↑	↑	Inflammation	[65, 79, 83, 90, 106, 123, 169, 194]
IL-18	↑	↑	Inflammation	[32, 42, 78, 137]
IL-6	↑	↑	Inflammation	[46, 57, 194]
HMGB1	↑	↑	Inflammation	[33, 149, 169, 180]
NFκB	↑	↑	Inflammation	[29, 101, 117, 169]
IL-8	↑	↑	Inflammation	[13, 45, 106, 123, 151, 156, 194]
IL-1β	↑	↑	Inflammation	[42, 46, 106, 122, 123]
IL-10	↑	↑	Anti-inflammatory	[81, 106, 123, 189]
iNOS	↑	↑	Oxidative stress	[90, 106, 123, 186]
Egr-1	↑	↑	Proliferation, tumorigenesis, apoptosis ferroptosis	[86, 154, 166, 181, 188]
PTEN	↑	↑	Pyroptosis	[87, 124, 178, 194]

## Molecular patterns of CME and cMI

### CME and cMI promote significant inflammatory responses

Post-infarction infiltration of monocytes, neutrophils, and macrophages is a key hallmark in both cMI and CME [132]. In cMI, neutrophil infiltration peaks between 1 and 3 days after MI [43]. During post-MI processes, the infiltrated monocytes and macrophages have been delineated to switch from a pro-inflammatory to an anti-inflammatory state [177]. Finally, in cMI, infiltrating pro-inflammatory Th1 lymphocytes were observed in porcine hearts within 48 h of reperfusion [48]. However, while the T-cell dynamics have been well documented in cMI, the T-cell repertoire warrants further investigation.

In the context of CME, infiltration of polymorphonuclear leukocytes has been observed in rats [7, 56, 63] and dogs [49]. These include neutrophils, monocytes, and macrophages, for which has been observed in rats [7, 104, 106, 123] and pigs [5, 152]. Of relevance, the highest density of leukocyte infiltration has been observed in the areas surrounding microthrombi while neighboring myocardial areas displayed significantly smaller infiltrates [80].

Alongside immune cell infiltration, other inflammatory biomarkers are known to be similarly upregulated in both cMI and CME. Specifically, in cMI, serum levels of TNF-*α* and PTEN were elevated in patients with acute ST-segment elevation MI (STEMI) [124]. In addition, cMI rat models have shown a strong upregulation of IL-1β and IL-6 mRNA 3–12 h post-ligation [46], alongside increased nuclear factor-κB (NF-κB) activation following IRI in cMI rats [29, 101]. Furthermore, increased systemic IL-8 expression was detected in patients with AMI [45, 151]. IL-8 has been associated with more complicated MI in STEMI patients, larger infarct area, reduced LVEF, and higher risk of MVO in PCI-treated STEMI patients [156]. Finally, significantly elevated levels of IL-18 were observed in a small cohort study of patients with AMI when compared to healthy subjects [137]. In a CME rat model, upregulation of the pro-inflammatory cytokines TNF-*α*, IL-1β and IL-10 was observed 3 h after injection [106, 123] alongside a decrease in LV ejection fraction (LVEF) [106, 123]. Immunohistological analysis of the microinfarct and surrounding area revealed edema and myocardial degeneration in addition to infiltration of monocytes and macrophages [123]. Induction of CME in pigs further identified monocytes and macrophages as a critical source of TNF-*α* [5], and upregulation of phosphatase and tensin homolog was positively associated with TNF-*α* expression [178]. In addition, high mobility group A1 (HMGA1) mRNA and NF-κB p65 protein expression were found to be significantly increased in a CME rat model, which was positively correlated with an increase in TNF-*α* expression [169]. This suggests an involvement of the HMGA1/NF-κB pathway in CME-induced myocardial injury [169]. In addition, CME induction in mini swine resulted in increased coronary sinus levels of IL-6, IL-8, and TNF-*α* [194]. While IL-6 and IL-8 levels returned to baseline, TNF-*α* levels remained elevated at the 30-day follow-up [194]. In addition, IL-18 was also found to be elevated in CME rats [32].

Collectively, despite significant differences in infarct size, these studies indicate the induction of a similar inflammatory pattern following both CME and cMI.

### CME and cMI have distinct inflammatory pathways and biomarkers

Despite these similarities, several studies comparing the systemic inflammatory profiles of CME-derived MINOCA and cMI reflect distinct underlying molecular mechanisms (Table 2). One important study, the PLATO trial, distinguishes biomarkers between MI and MINOCA patients. When comparing the expression of several biomarkers in the acute phase of MINOCA and cMI patients, for instance, the concentration of high-sensitivity C-reactive protein (hs-CRP) was increased in MINOCA compared to cMI [70], however, with comparable levels of N-terminal pro-B-type natriuretic peptide (NT-proBNP), suggesting a similar degree of myocardial dysfunction in MINOCA and cMI [70]. Comparison of plasma samples from patients 3 months after MINOCA or cMI and healthy controls further revealed a persistent pro-inflammatory activity in MINOCA patients with higher levels of NF-kappa-B essential modulator (NEMO), IL-6 and soluble urokinase plasminogen activator receptor (suPAR) [71]. In addition, compared to cMI patients, MINOCA patients exhibited higher levels of P-selectin glycoprotein ligand-1 (PSGL-1), C-X-C motif chemokine ligand 1 (CXCL1), TNF-related activation-induced cytokine (TRANCE) and lower levels of interleukin-1 receptor antagonist (IL-1RA), further suggesting distinct inflammatory signaling following MINOCA [71]. Since MINOCA affects more female patients, whole blood transcriptome profiling of women suffering from either MINOCA or cMI in addition to healthy controls had been performed to dissect potential differences. This identified estrogen receptor pathways, mechanistic target of rapamycin (mTOR), and E74-like factor 2 (ELF2) signaling as the top differentially expressed pathways in MINOCA [12]. In addition, a decrease in transcript levels of members of the phosphoinositide 3-kinase (PI3K) pathway and an increase in transcripts associated with cytotoxic T cells were observed, further highlighting the presence of distinct inflammatory processes underlying MINOCA [12].

**Table 2:** Differentially expressed markers in MINOCA and cMI. *TUG1* taurine upregulated gene 1, *RhoGDI α* rho GDP-dissociation inhibitor 1, *tPA* tissue plasminogen activator, *PSGL1* P-selectin glycoprotein ligand-1, *CXCL1* C-X-C motif ligand 1, *TRANCE* tumor necrosis factor-related activation-induced cytokine, *PAPPA* pregnancy-associated plasma protein-A, *IL* interleukin, *MPO* myeloperoxidase, *BNP* B-type natriuretic peptide

Marker	MINOCA/CME	cMI	Cellular process	References
TUG1	↓	↑	Fibrosis, pyroptosis	[168, 182, 199, 200]
RhoGDI*α*	↓	–	Apoptosis, proliferation, migration	[30]
miR-30e-3p	↓	–	Autophagy	[42, 199]
miR-186-5p	↑	–	Cell proliferation, apoptosis, angiogenesis (in cancer)	[183]
tPA	–	↑	Coagulation, fibrinolysis	[71, 113]
Filamin A	↓	–	Cytoskeleton	[30, 197]
PSGL1	↑	–	Inflammation	[8, 71]
CXCL1	↑	–	Inflammation	[71, 187]
TRANCE	↑	–	Inflammation	[71]
PAPPA	↑	–	Inflammation	[71, 108]
IL-1 RA	–	↑	Anti-inflammatory	[4, 71]
MPO	–	↑	Oxidative stress, inflammation	[71, 134]
BNP	–	↑	Vasodilatation, RAAS inhibition	[71, 184]

Moreover, clinically established biomarkers of MI have been shown to follow distinct patterns in cMI and MINOCA. cMI patients had elevated levels of tissue-type plasminogen activator (tPA) compared to MINOCA, which were comparable to control levels [71]. In addition, cMI patients exhibited higher levels of B-type natriuretic peptide (BNP) and myeloperoxidase (MPO) than MINOCA and control patients—both markers of cardiac dysfunction [71]. A study showed that while MINOCA appeared to be independent of glucose levels, obstructive AMI patients were more often hyperglycemic with changes in neutrophil-to-lymphocyte ratio, neutrophil-to-platelet ratio, and platelet-to-lymphocyte ratio [140].

Most recently, systemic molecular patterns of cMI and MINOCA have been compared in a translational porcine model mimicking all clinical aspects of cMI and MINOCA. Hereby, MINOCA was associated with significantly increased levels of leukotriene-A4-hydrolase and its enzymatic product leukotriene B4 indicating a distinct upregulation of the leukotriene pathway in these animals. Notably, PBMCs isolated from MINOCA animals and subjected to leukotriene B exhibited an augmented inflammatory cytokine secretion including TNF-*α*. These observations translated into comparable levels of pro-inflammatory cytokines including TNF-*α*, IL-1*α* and IL-1β in groups despite significantly smaller infarction areas in animals undergoing MINOCA [80].

**Fig. 4:**
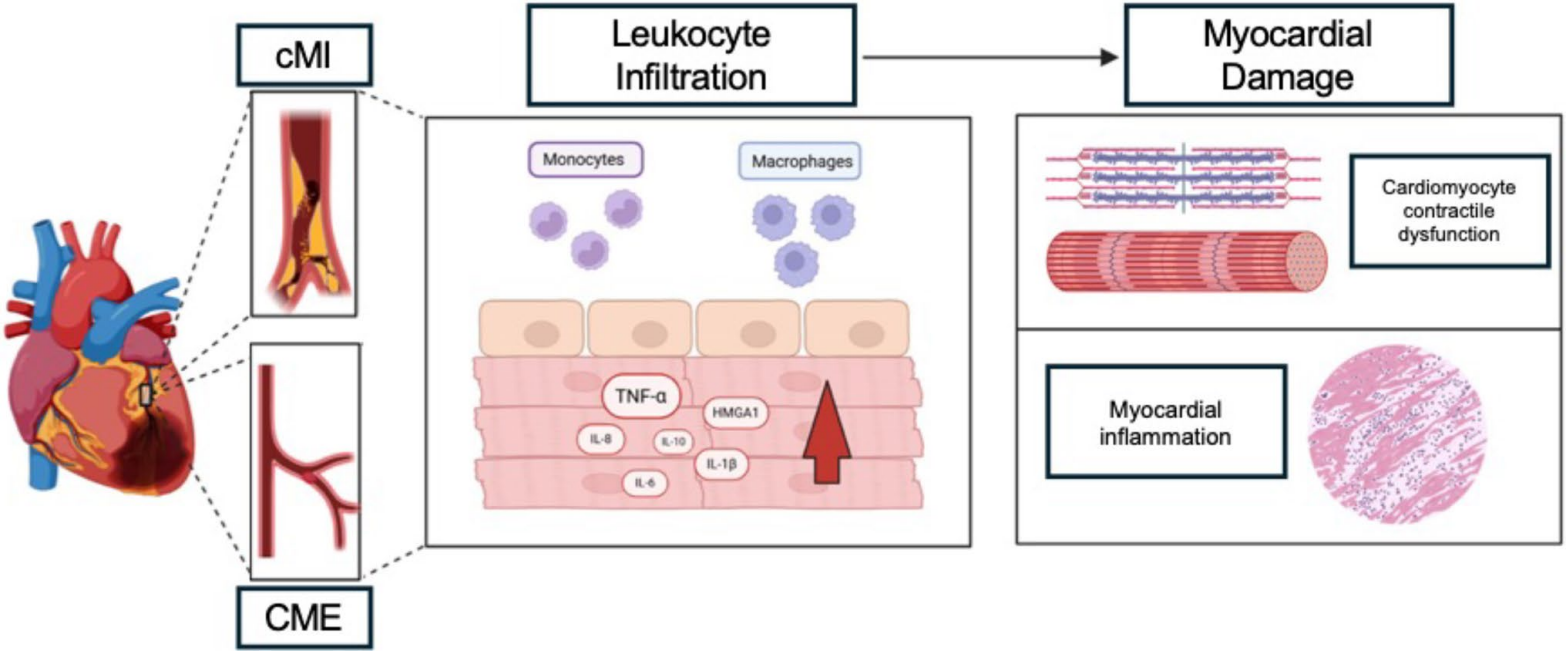
Inflammatory pathways implicated in cMI and CME. Both cMI and CME share key pathological features, including robust leukocyte infiltration, comparable patterns of myocardial inflammation, evidence of myocardial damage, and resulting contractile dysfunction, despite differences in infarct size. *cMI*, classic myocardial infarction; *CME*, cardiac microembolization. Created with *BioRender.com*

In summary, these studies suggest distinct pathways instigated through CME and cMI to promote tissue inflammation and cardiac dysfunction, which may also serve as potential therapeutic targets (Fig. 4).

### CME and cMI induce similar patterns of cellular death and tissue damage

**Fig. 5:**
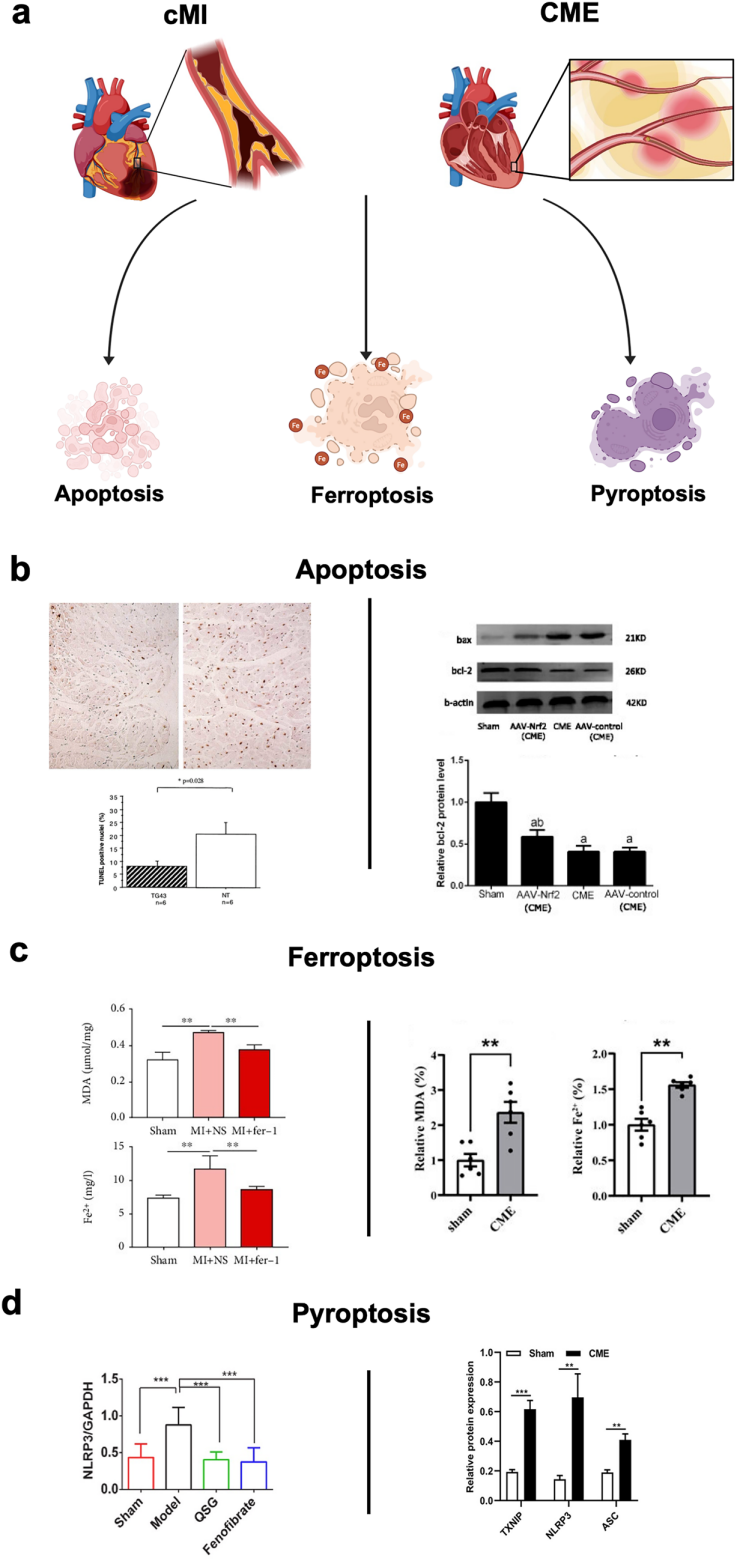
Cell death mechanistic comparisons in MI and CME-induced MINOCA models. **a** Overview of various cell death mechanisms occurring in both MI and CME-induced MINOCA. Created with *BioRender.com*. **b** Comparison of apoptosis occurring in MI and CME models. The left hand panels were reproduced with permission from Brocheriou et al*.* [21] (Wiley, 2000). Brocheriou et al. [21] show TG43 Bcl-2 (*n* = 6 biological replicates) and non-transgenic animals (*n* = 6 biological replicates) with representative TUNEL analysis of the infarct border zone of two representative animals. All data from Brocheriou et al. [21] were subjected to ANOVA followed by Fisher's t test and a *p* value of < 0.05 was considered significant. Right hand side panels were reproduced under terms of the CC-BY license from Liang et al. [110] (Springer Nature, 2017). Data from Liang et al. [110] were shown as Western blot bands (Bax, Bcl-2, and B-actin) for sham animals, adeno-associated virus (AAV)-Nrf2 with coronary microembolization (CME), CME alone, and AAV-control, with Bcl-2 protein levels analyzed and represented as mean ± SD. **a** P < 0.05 contrasted with sham; **b** *P* < 0.05 contrasted with CME or AAV-control (CME). **c** Comparison of ferroptosis occurring in MI and CME models. The left hand panels were reproduced under terms of the CC-BY license from Li et al. [107] (Wiley, 2021). Evaluation of malondialdehyde (MDA) and Fe2+ levels in infarcted myocardial tissue. Upper: MDA level; bottom: Fe2 + level. Pooled data: biological replication indicated *n* = 5 mice per group. Data were presented as mean ± SEM. **P < 0.01 compared with sham by one-way ANOVA with Bonferroni’s post hoc test. The right hand panels were reproduced under terms of the CC-BY license from from Liu et al. [116] (Frontiers, 2022). Cardiac levels of GSH, Fe2+, and MDA were measured (*n* = 6). All data were presented as mean ± SEM, with student’s t-test applied to assess variations between two independent groups (*p < 0.05 and **p < 0.01). **d** Comparison of pyroptosis occurring in MI and CME models. The left hand panels were reproduced with permission from Chen et al. [34] (Elsevier, 2002). NLRP3 immunoblots were quantified between sham, model, qishen granule (QSG), and fenofibrate (*n* = 4 per group). ***P < 0.001 vs. model group through one-way ANOVA, with data displayed as mean ± SD. The right hand panels were reproduced under terms of the CC-BY license from Chen et al. [36] (Frontiers, 2021). Expression levels of proteins related to cardiomyocyte pyroptosis (*n *= 3 per group). The data are presented as the mean ± standard deviation (SD). *P < 0.05, **P < 0.01, ***P < 0.001, done through one-way ANOVA

Besides histological and cytokine changes, both CME and cMI have also been shown to induce similar apoptotic pathways (Fig. 5). In a porcine model in which microspheres were injected into the left anterior descending artery (LAD) to induce CME, increased levels of lectin-like oxidized low-density lipoprotein receptor-1 (LOX-1), Bcl-2 associated X-protein (BAX), cytochrome c, and cleaved caspase-3, -8, and -9 were detected [118]. This suggests that CME induces cardiomyocyte apoptosis through the LOX-1-dependent pathway in the mitochondria as well as the caspase-8-dependent pathway [118]. In mice, LOX-1 expression was increased after ischemic injury and induced cardiomyocyte apoptosis [11]. A rat model of CME also showed a decrease in the expression of Bcl-2 [110] a key anti-apoptotic protein [93]. Similarly, findings suggest a critical role of Bcl-2 dysregulation in MI, with a direct association of Bcl-2 expression with cardiomyocyte apoptosis and heart function with upregulation of Bcl-2 Associated X-protein and caspase-3, and downregulation of Bcl-2 after MI [93].

Ferroptosis, another form of programmed cell death, has also been implicated in CME and cMI [116]. This form of cell death is initiated by iron-dependent lipid peroxidation leading to mitochondrial membrane condensation [76, 172] and can spread rapidly from one cell to another [172]. Initiated by mitochondrial ROS production, ferroptosis propagates through lipid peroxidation [172]. After induction of CME in rats, the expression of several ferroptosis markers, such as glutathione peroxidase 4, was decreased, whereas levels of iron and malondialdehyde were increased [116]. Notably, iron overload has been found in STEMI patients with associated adverse cardiac remodeling [22]. In addition, treatment with ferrostatin-1, an inhibitor of ferroptosis, reduced infarct size in cMI mice [107]. Interestingly, a study examining various cell death pathways 40 days after cMI in a rat model found evidence of apoptosis and pyroptosis, but not ferroptosis [145].

In addition, the same study further suggested pyroptosis as the dominant mode of programmed cell death after cMI [145]. Pyroptosis is a form of cell death characterized by cell swelling and membrane rupture, a release of pro-inflammatory cytokines and subsequent inflammatory response [171], directly linking cell death to inflammation [59]. Notably, pyroptosis has been implicated in both cMI and CME through activation of the NOD-, LRR- and pyrin domain-containing protein 3 (NLRP3)/caspase 1 pathway [36, 103, 190], with augmented caspase 1 levels in both infarction lesions [34, 36]. High levels of adenosine triphosphate and mitochondrial ROS, similar to those seen in ferroptosis, have been shown to activate NLRP3 and thus induce pyroptosis [35, 84].

### miRNAs and lncRNAs play a significant role in CME injury and inflammation

**Fig. 6:**
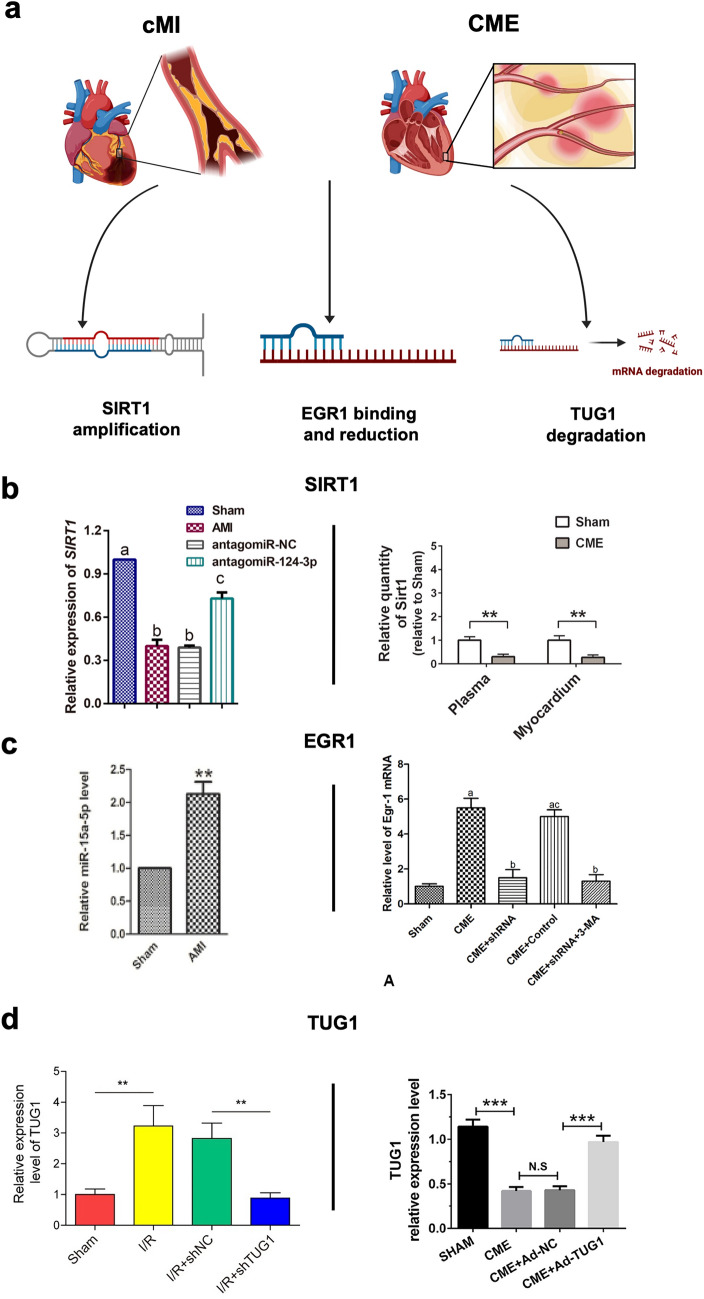
Impact of targeting miRNAs in mediating Inflammation in MI and CME-induced MINOCA models. **a** Overview of unique conserved miRNA targeting mechanisms occurring in both MI and CME-induced MINOCA. Created with *BioRender.com*** b** Comparison of SIRT1 amplification to mediate negative outcomes in MI and CME models. The left hand panel was reproduced with permission from Wei et al*.* [185] (Springer Nature, 2021). The right hand panel was reproduced with permission from Gao et al. [61] (Elsevier, 2022). Sirt1 mRNA expression was evaluated in the plasma and myocardium of rats through RT-qPCR with *n* = 12 per sham and CME. **P < 0.01 by one-way ANOVA. **c** Comparison of EGR1 binding and reduction occurring in MI and CME models. The left hand panel was reproduced with permission from Fan et al. [55] (Elsevier, 2021). The mRNA level of miR-15a-5p was measured by qRT-PCR. *n* = 6. **P < 0.01 vs. sham group, evaluated by two-tailed Student’s t-test. The right hand panel was reproduced under terms of the CC-BY license from Wang et al. [181] (Aging-US, 2018). Egr1 mRNA was evaluated in rat myocardium via RT-qPCR. Results are presented as mean ± SD (*n* = 3 technical replicates). **a** *P* < 0.05 compared with the sham group; **b** *P* < 0.05 compared with the CME group; **c** *P* < 0.05 compared with the CME + shRNA group, via one-way ANOVA. **d** Comparison of TUG1 levels in MI and CME models. The left hand panel was reproduced under terms of the CC-BY license from Wang et al. [182] (Springer Nature, 2022). TUG expression was measured using qRT-PCR in the myocardial tissue of I/R mice (*n* = 8 per group). **P < 0.01, with one-way ANOVA performed with Tukey’s post hoc test for comparisons between each group. The right hand panel was reproduced under terms of the CC-BY license from Zhou et al. [199] (Frontiers, 2021). Transfection with AAV-pcDNA3.1-TUG1 significantly increased myocardial TUG1 level following CME (*n* = 10). Data are mean ± SEM from three or more independent experiments. *P < 0.05, **P < 0.01, ***P < 0.001, evaluated via one-way ANOVA followed by the Bonferroni test

Recent studies have delineated several microRNAs (miRNAs), which are small single-stranded non-coding RNAs, and long non-coding RNAs (lncRNAs), as crucial drivers of cardiac injury, inflammation and regeneration following myocardial infarction, with potential as diagnostic biomarkers [41, 62, 198] (Fig. 6). Further underscoring the implication of miRNAs in the pathophysiology of CME, GO and KEGG analyses have shown upregulation of a broad range of miRNAs associated with necrosis, apoptosis, inflammation, and fibrosis [167]. Therefore, there has been further investigation into the implication of miRNAs in CME-derived tissue injury and inflammation.

One notable example is miR-29b-3p, which is implicated in myocardial ischemia/reperfusion injury (IRI) [115] and subsequent cardiac fibrosis [176]. Following microsphere injection in a rat CME model, miR-29b-3p was found to be upregulated, leading to a reduction in myocardial apoptosis, enhanced angiogenesis and improved cardiac function [148]. Mechanistically, miR-29b-3p has been shown to disrupt TNF-*α* signaling by suppressing TNF receptor-associated factor 5 and thus protecting cardiomyocytes from hypoxia-induced apoptosis [23]. Moreover, miR-29b-3p targets glycogen synthase kinase-3 β and Bcl2-modifying factor, two key apoptotic markers, thus mitigating apoptosis regulation resulting from CME [148].

Another CME-related miRNA is miRNA-30e-3p, which has been downregulated post-microthrombi injection in rats. miRNA-30e-3p downregulation has been linked to augmented inflammasome activation, higher IL-18 and IL-1β expression, and leukocyte infiltration that thus translate into cardiac function decline [42]. Overexpression of miR-30e-3p, in turn, dampened inflammasome signaling, reduced microinfarcts, and restored cardiac function [42]. Consistently, reduced miR-30e-3p expression was associated with decreased autophagy following exposure to prolonged hypoxia with augmented cardiomyocyte apoptosis [166]. Mechanistically, miR-30e-3p has been shown to regulate early growth response 1 (Egr-1) activation. Egr-1 has been suggested to impair cardiac function following CME by regulating autophagy and apoptosis in rats [181] and mice [55].

Finally, SIRT1 signaling has been noted to act on multiple miRNAs to induce overall protection in cardiovascular disease. SIRT1 has been shown to regulate endothelial function, inhibit platelet aggregation, and attenuate vascular inflammation [9, 27]. Notably, different regulatory pathways of SIRT1 have been uncovered in MINOCA and cMI. After CME, SIRT1 was inhibited through upregulation of miR-34-5p, which has been shown to be a discriminator of prethrombotic status in patients with stable cMI [60]. SIRT1 reduction was found to enhance microthrombosis and pronounce cardiac dysfunction [61]. In cMI in turn, miR-124-3p mediated downregulation of SIRT1 and thus led to increased production of pro-inflammatory cytokines, such as IL-1β, TNF-*α* and IL-6, alongside increased oxidative stress and apoptosis [185].

Serum miRNA profiling in animals undergoing cMI and MINOCA also indicated that altered tissue expression of miRNAs translates into differential systemic profiles. Animals in the cMI group exhibited increased expression of miRNAs associated with myocardial ischemia and reperfusion, including ssc-mir-139-5p, ssc-mir-18a [112] and ssc-miR-30 [201]. In contrast, MINOCA induced a more heterogeneous miRNA profile, with altered expression of ssc-mir-615 and ssc-mir-484, as well as downregulation of ssc-mir-145-5p. Moreover, differential expression analysis revealed significant upregulation of ssc-mir-330, ssc-mir-105, ssc-mir-26a, ssc-mir-199-3p, and ssc-mir-92a, all of which are related to cardiac IRI in cMI animals. In MINOCA animals, ssc-mir-802 was upregulated compared to MI counterparts [80].

In addition to miRNAs, studies investigating the role of the long non-coding RNAs taurine upregulated gene 1 (TUG1) in CME and MI reported inverse regulation with distinct signaling pathways leading to inflammation. Following CME, TUG1 was significantly downregulated leading to an accelerated NLRP3-inflammasome activation with augmented pro-inflammatory IL-1β and IL-18 production and pyroptosis [199]. Mechanistically, TUG1 downregulation abolished the inhibitory effects on miRNA-186-5p, which in turn accelerates inflammasome activation by directly inhibiting X-linked inhibitor of apoptosis protein [199]. In contrast, following cMI, hypoxia, and reoxygenation increased TUG1 expression directly through hypoxia-inducible factor 1-alpha signaling, which ultimately also promotes NLRP3 inflammasome activation [182]. In line with these observations, other studies reported increased TUG1 expression after cMI [195] and IRI [168], which exacerbated myocardial injury and fibrogenesis through sponging distinct miRNAs.

### Biomarkers of microvascular occlusion after PCI may differentiate cMI and CME-induced MINOCA

Percutaneous coronary intervention (PCI) in patients with coronary artery disease constitutes a clinically relevant human model for MINOCA. Typically, patients with stent deployment undergo mechanical disruption of atherosclerotic plaques, resulting in distal embolization of either thrombotic or atherosclerotic material into the coronary microcirculation [66]. Through intravascular ultrasound and optical coherence tomography studies, plaque morphological features have been characterized by a large necrotic core volume, thin fibrous cap architecture, and lipid content, all of which are principal determinants of embolic burden post-PCI [6, 17]. From a diagnostics standpoint, myocardial blush grade, index of microcirculatory resistance, and thrombolysis in myocardial infarction flow grade are all obtained during PCI, serve as surrogate markers of MVO severity, and correlate with downstream biomarker profiles [69]. In total, PCI provides a human analog of CME where biomarker discovery, plaque characterization, and therapeutic intervention can be studied with temporal prediction, enabling further use in MINOCA biomarker studies.

As microvascular obstruction (MVO) due to embolization has been shown to cause MINOCA and is an important complication after PCI in both STEMI and NSTEMI patients [47], these specific biomarkers referenced throughout this manuscript may be valuable in guiding biomarker discovery and diagnostic strategies for MINOCA. Since MVO after PCI causes the no-reflow phenomenon [82] similar to CME [14], the identified biomarkers may also be applicable to MINOCA and therefore warrant further investigation.

In the context of MVO, several biomarkers have been identified to help understand whether a patient is at risk for MVO post-PCI. In a study of patients undergoing primary PCI for STEMI, post-procedural C-reactive protein (CRP) and troponin levels were elevated in patients who experienced MVO [77]. In addition, increased serum levels of thrombomodulin, lymphatic vessel endothelial hyaluronan receptor-1, and syndecan-1 were observed [77]. CRP, troponin and syndecan-1 levels were positively associated with the risk of MI after PCI [77]. Another study showed that STEMI patients who experienced MVO after primary PCI had significantly higher levels of CRP, fibrinogen, and neutrophils [16]. Other biomarkers found to be associated with MVO after PCI include elevated levels of creatine kinase, hsCRP, lactate dehydrogenase, aspartate aminotransferase, and alanine aminotransferase [47]. This suggests a possible association with inflammatory conditions [184] that may be similar to those seen after CME-induced MINOCA.

miRNAs play a critical role in MVO pathophysiology. For example, elevated levels of miR-1 and miR-133b were positively associated with MVO after PCI [39]. Dysregulation of miR-133b levels in STEMI patients has been shown to be associated with a higher risk of death [40]. In addition, miR-133b expression was elevated in STEMI patients compared to NSTEMI patients [95]. The cardioprotective properties of miR-133b have been demonstrated by inhibiting apoptosis in morphine-preconditioned rat cardiomyocytes [64]. Similarly, overexpression of miR-133b in HL-1 cardiomyocytes prevented apoptosis and suppressed the accumulation of collagen under doxorubicin treatment [109]. In addition, AAV-mediated overexpression of miR-133b in mice attenuated cardiac fibrosis by decreasing the deposition of collagen [109]. Interestingly, patients with an occluded infarct-related artery were reported to have higher levels of miR-133b than patients with patent infarct-related coronary arteries [58].

Collectively, these findings suggest that a significant pathological response is instigated through CME, which promotes a distinct immune response, cellular death, tissue injury, specific miRNA response and ultimately cardiac dysfunction.

### CME modifies the efficacy of cardioprotective drugs

Finally, the interactions between CME and established cardioprotective mechanisms have been largely overlooked in treating CME pathophysiology. Both ischemic preconditioning and postconditioning have been performed in cMI to limit cardiomyocyte death upon reperfusion, specifically through the reperfusion injury salvage kinase and survivor activating factor enhancement pathways [158, 164, 165]. However, concurrent CME has been shown to attenuate their protective efficacy with a mechanistically complex relationship. Prior microembolization has been shown to release adenosine concentrations into the coronary vasculature, but it cannot raise adenosine concentrations interstitially to activate preconditioning [164], demonstrating that CME does not confer ischemic preconditioning cardioprotection. However, when preconditioning is applied after CME, infarct size is reduced but exclusively in non-microembolized tissue, creating the appearance of failed protection when evaluated globally [158]. Here, spatial heterogeneity provides an explanation for the apparent cardioprotection attenuation following CME.

In comparison, the relationship between ischemic postconditioning and CME is particularly relevant to the clinical PCI setting, as unintentional microembolization occurs post-PCI and at reperfusion, allowing microemboli to extend infarct size and augment border zone injury [165]. Here, postconditioning remains protective even in the presence of concurrent CME by reducing infarct size and preventing embolic accumulation at the border zone, suggesting that postconditioning targets the same reperfusion-phase injury exacerbated by CME. Clinically, these data suggest that microembolization post-PCI leads to limited benefit in large clinical trials despite promising preclinical evidence. In addition, TNF-*α* has been shown to exhibit a bidirectional effect in CME by driving progressive contractile dysfunction while providing delayed protection against infarction through cardioprotective pathways [159]. In short, these findings suggest that CME thus shifts the spatial and temporal regimes of cardioprotective mechanisms and that anti-CME interventions could be paired with postconditioning to limit MINOCA reperfusion injury.

## Investigation of molecular mechanisms of MINOCA could pave the way for novel diagnostic and therapeutic opportunities

As there are no MINOCA-specific therapies available, further investigation and testing of molecular therapies targeting biomarkers found in CME-induced MINOCA. Small molecular therapies have been used to treat CME-induced MINOCA by reducing cellular death, reducing inflammation and improving cardiac function. Glycyrrhizin, a high mobility group box 1 (HMGB1) inhibitor, significantly lessened oxidative stress and apoptosis alongside reducing inflammation in a CME model. It also reduced various inflammatory markers including TNF-*α* and iNOS [192], improved cardiac function, and inhibited CME-induced cardiomyocyte apoptosis [192]. Moreover, alprostadil, a small molecule inhibitor of platelet aggregation, was able to improve microcirculation flow and vasodilation [147] in a CME setting. Alprostadil administration in a CME rat model significantly reduced oxidative stress, myocardial apoptosis and improved cardiac function [147]. Another drug that has been studied in a CME setting is nicorandil, a nicotinamide nitrate and donor of NO that has also shown protective effects following PCI with significantly improved cardiac function and reduced myocardial damage [170]. Notably, in rats, nicorandil treatment significantly prevented CME-induced inflammation through inhibition of toll-like receptor 4-mediated signaling [170] and the NLRP3 inflammasome, leading to improvement in cardiac function, alleviation of myocardial injury, and attenuation of oxidative stress [119].

In addition, with the knowledge that cMI can exhibit similar phenotypes to CME-induced MINOCA, repurposing cMI treatments could be another avenue toward treating CME-induced MINOCA. Statins, commonly used to treat cardiovascular disease, have also shown clinical benefit for MINOCA studies. A systematic review and meta-analysis for statin use in MINOCA showed that statin treatment reduced the number of major cardiovascular events as well as mortality [121, 126]. Mechanistically, statin premedication reduced ROS levels [31], diminished cell death [105, 179], attenuated inflammatory responses [116, 179], and ultimately preserved myocardial [116] damage and improved cardiac function [105, 179].

**Fig. 7:**
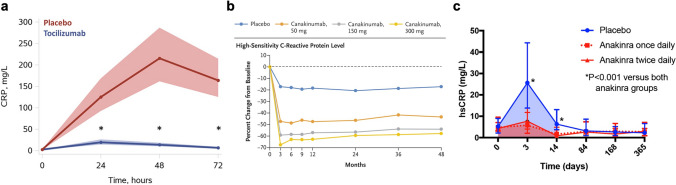
Anti-inflammatory antibodies restrict the inflammatory response following MI and improve outcomes in clinical trials. **a** CRP (C-reactive protein) levels for tocilizumab (*n* = 39) vs. placebo (*n* = 41) from the IMICA trial. Results are presented as means with 95% confidence intervals based on predicted values from linear mixed-model analysis with baseline correction (PROC MIXED, SAS Institute Inc.). *P ≤ 0.0001. Reproduced under the CC-BY-NC-ND license from Meyer et al*.* [129] (Wolters Kluwer Health, 2021). **b** CRP levels for canakinumab 50 mg (*n* = 2170), canakinumab 150 mg (*n* = 2284), canakinumab 300 mg (*n* = 2263) vs. placebo (*n* = 3344). P < 0.001 for all comparisons of the median percentage change in a canakinumab group with the placebo group. Reproduced with permission from Ridker et al. [150] (NEJM, 2017). **c** CRP levels for anakinra once daily (*n* = 33) vs. anakinra twice daily (*n* = 31) vs. placebo (*n* = 35). Reproduced under the CC-BY-NC-ND license from Abbate et al. [1] (Wiley, 2020). *P* < 0.001 for each anakinra group vs. placebo, and *P* < 0.001 for anakinra groups combined vs. placebo. The differences between treatment groups were computed using the Wilcoxon or Kruskal–Wallis rank‐sum test for two groups (anakinra [both groups] versus placebo) or three groups, respectively, for continuous variables. All values were reported as the median and interquartile range for potential deviation from Gaussian distribution

Given inflammatory similarities between CME-induced MINOCA and cMI, targeting the inflammatory response is another promising avenue for treating CME-derived MINOCA. Antibody therapies targeting lead cytokines including IL-6, IL-1 and TNF-*α* have hereby shown significant effects on reducing cardiac inflammation in cMI (Fig. 7).

Tocilizumab, an IL-6 receptor antagonist, has been shown to attenuate inflammation after MI in STEMI patients and improve clinical outcomes [127] with an increased myocardial salvage index assessed by MRI 3–7 days post-PCI [20]. Moreover, tocilizumab also reduced the inflammatory response following NSTEMI with significantly reduced cardiac troponin levels post-PCI [92]. Interestingly, tocilizumab treatment also reduced systemic inflammation and myocardial damage in comatose patients resuscitated from out-of-hospital cardiac arrest [129].

Canakinumab, a monoclonal antibody targeting IL-1β, was administered to patients with a history of cMI and increased CRP levels. This treatment elicited a reduction in systemic CRP levels linked to reduced adverse events such as cardiovascular death, MI or stroke [25]. However, this was associated with an increased risk of fatal infections [25]. Blocking the IL-1 receptor with the recombinant receptor antagonist anakinra, in turn, dampened CRP levels following MI and reduced mortality and heart failure in a randomized controlled trial [1]. Lastly, TNF-*α* inhibition with Infliximab demonstrated therapeutic value in an experimental setting, improving left ventricular ejection fraction and reducing scar size in a porcine model of MI [120]. Supporting a therapeutic value also for MINOCA, treatment with neutralizing TNF-*α* antibodies in a dog model of CME annihilated contractile dysfunction and improved posterior wall thickening [50]. Since CME has been associated with a similar systemic inflammatory cytokine profile, these antibodies may also have therapeutic potential for CME and MINOCA.

Finally, the anti-inflammatory potential of corticosteroids has been explored preclinically. In a preclinical CME model, cortisone administration reduced the inflammatory response and leukocyte infiltration, alongside preserving contractile function relative to untreated controls [161]. Cortisone treatment demonstrated NF-κB dependent suppression, leading to downstream suppression of TNF-*α*, IL-1β, and IL-6, all of which are upregulated post-CME. While the clinical use of corticosteroids remains controversial for acute MI due to risk of myocardial rupture [96], the microinfarct pattern within CME-induced MINOCA may present a more favorable risk profile. Thus, corticosteroids or their derivatives could be viable for use in treating the specific inflammatory profile found in MINOCA.

Considering the observation of augmented leukotriene signaling in pigs undergoing MINOCA [80], repurposing leukotriene receptor antagonists may be of therapeutic value. Indeed, inhibiting leukotriene signaling during cMI by Montelukast [131] has been shown to impede excessive inflammation, reduce TNF-*α* and IL-1β expression, diminish leukocyte infiltration, and improve cardiac function while preventing maladaptive remodeling in experimental models of MI [74, 131].

As mentioned previously, targeting miRNAs could be significant for CME-induced MINOCA. For instance, upregulation of miR-200a-3p via transfection with an adeno-associated virus vector resulted in inhibition of cardiomyocyte apoptosis and no myocardial damage after CME through inhibition of the NLRP3 pathway [36]. Silencing of the previously mentioned miRNA miR-34a-5p, in turn limited myocardial inflammation and resulted in a decrease in the observed apoptosis following CME [60]. On the other hand, overexpression of miRNAs with cardioprotective properties such as miR-29b-3p was shown to decrease apoptosis, promote angiogenesis, and improve cardiac function [148].

## Conclusions

Given that MINOCA and cMI have similar presentations, many patients with MINOCA are misdiagnosed and standard therapy is initiated late, resulting in a mortality rate comparable to cMI despite less myocardial damage. Although few studies have directly compared MINOCA and cMI, many studies are investigating the molecular mechanisms behind CME-mediated MINOCA pathologies. While clinical presentation and histology demonstrate differences between MINOCA and cMI, these models demonstrate a conserved inflammatory response despite significantly different infarct sizes for both CME-derived MINOCA and cMI. However, the molecular pathways driving the inflammatory response, cellular death, and miRNA presentation were distinct, which may allow discrimination and targeted therapy of the two entities. As a result of finding these biomarkers, preclinical and clinical studies already demonstrate the feasibility of targeting these biomarkers for MINOCA treatment. Future studies should focus on dissecting further molecular differences in the pathophysiology of cMI and MINOCA to identify discriminative biomarkers. By leveraging unique immune, cell death, and miRNA biomarkers, clinicians can use this knowledge for diagnostic purposes and to enable biomarker-based tailored therapy development for MINOCA. This review reveals multiple potential biomarkers that must be evaluated for differential levels of expression if they are elevated in both cMI and MINOCA. If they are differentially regulated, they must be validated in a clinical setting. If proven true, new biomarkers may be discovered and integrated into the initial patient admission process through a blood draw and subsequent biomarker level determination using methods such as an enzyme-linked immunosorbent assay. Ultimately, the right diagnosis will enable patients to receive appropriate treatment and reduce the high MINOCA mortality rate.

## Data Availability

The data supporting the findings of this study are derived from the articles listed in the References section. No new datasets were generated or analyzed for this study.
